# Proteinuria After Kidney Transplantation

**DOI:** 10.1111/petr.70233

**Published:** 2025-11-24

**Authors:** Tomas Seeman, Robert L. Myette, Janusz Feber, Massimiliano Bertacchi, Raja Sekhar Dandamudi, Daniella Levy Erez, Anja Buescher

**Affiliations:** ^1^ Department of Pediatrics, Second Faculty of Medicine Charles University Prague Czechia; ^2^ Department of Pediatrics University Hospital Ostrava and Medical Faculty University of Ostrava Ostrava Czechia; ^3^ Children's Hospital of Eastern Ontario Research Institute & Children's Hospital of Eastern Ontario, Division of Pediatric Nephrology University of Ottawa Ottawa Ontario Canada; ^4^ Kidney Research Center, Ottawa Hospital Research Institute University of Ottawa Ottawa Ontario Canada; ^5^ Division of Pediatric Nephrology, Department of Pediatrics, Gynecology and Obstetrics Geneva University Hospitals Geneva Switzerland; ^6^ Division of Pediatric Nephrology Washington University in St. Louis School of Medicine St. Louis Missouri USA; ^7^ Schneider Children's Medical Center Petach Tiqva Israel; ^8^ Faculty of Health Sciences Tel Aviv University Tel Aviv Israel; ^9^ Children's Hospital of Philadelphia Philadelphia Pennsylvania USA; ^10^ Department of Pediatrics II, University Hospital of Essen University of Duisburg‐Essen Essen Germany

**Keywords:** angiotensin‐converting enzyme inhibitors, blood pressure, children, graft survival, hypertension, kidney transplantation, proteinuria

## Abstract

Proteinuria is a relatively frequent complication in both adults and children after kidney transplantation (40%–80%). It is usually mild and predominantly of tubular origin and is caused mainly by rejection, mTOR inhibitors, or hypertension; however, proteinuria could also be in the nephrotic range and of glomerular origin if caused by the recurrence of idiopathic FSGS or rejection. Proteinuria is a risk factor impacting graft and patient survival in adults and graft survival in children. Proteinuria should be assessed by protein/creatinine ratio regularly in pediatric kidney transplant recipients. In children with idiopathic FSGS, proteinuria should be assessed daily during the first 2–3 weeks post‐transplant to enable prompt diagnosis of recurrence. The etiology of proteinuria should be identified (recurrence, rejection, mTOR‐inhibitors, hypertension, etc.). If no apparent cause is found, a graft biopsy should be considered. Antiproteinuric therapy is primarily focused on treating the causes of the proteinuria, and this is usually done using Angiotensin‐converting enzyme inhibitors (ACEI) and angiotensin receptor blockers (ARBs). The long‐term follow‐up goal should be normalization of proteinuria with a protein/creatinine ratio < 20 mg/mmol (200 mg/g). Because of the role elevated blood pressure may play in exacerbating proteinuria, antihypertensive medications should be used in those who are resistant to initial antiproteinuric therapy to achieve lower BP.

**Abbreviations**
ACEI: angiotensin converting enzyme inhibitor
ARB: angiotensin receptor blocker
BKV: BK polyomavirus
BP: blood pressure
CKD: chronic kidney disease
CMV: cytomegalovirus
EBV: Epstein Barr virus
ESCORT: trial Effects of strict control of blood pressure in pediatric renal transplant recipients
ET: endothelin
FSGS: focal segmental glomerulosclerosis
KDIGO: kidney diseases improving global outcomes
MRA: mineralocorticoid receptor antagonists
mTOR: mammalian target of rapamycin
RAAS: renin‐angiotensin‐aldosterone system
SGLT2: sodium‐glucose‐contrasporter‐2
TG: transplant glomerulopathy

## 1 Introduction

Proteinuria is a common complication in patients with chronic kidney disease (CKD). Combined with arterial hypertension, these are the most important risk factors for progression of chronic kidney disease and GFR decline [1, 2, 3]. In the last decade there has been a growing body of literature focused on the role of proteinuria in adult and pediatric kidney transplant recipients as a treatable risk factor for graft loss and patient mortality [4].

Our review summarizes the approach to evaluation and management of proteinuria in patients after kidney transplantation. It will review the measurement of proteinuria, the prevalence of post‐transplant proteinuria, timing, and types of proteinuria, as well as causes. Furthermore, we will describe for the readers the known clinical consequences of proteinuria for post‐transplant patients, in relation to both overall survival and graft survival. Lastly, we will examine treatment approaches, both old and new, and propose areas for future research. Overall, we aim to provide a comprehensive assessment of proteinuria in patient's post‐kidney transplant.

## 2 Measurement of Proteinuria

A semi‐quantitative analysis using urinary dip‐stick is a suitable method to detect proteinuria in the outpatient setting. However, exact quantification is needed to decide whether further diagnostics (e.g., graft biopsy) are necessary, and to plan necessary follow‐up monitoring. A 24 h urine collection is considered to be the gold standard to obtain reliable results. However, collection of urine across a 24 h period is challenging, especially in small children with day (and/or night) incontinence. As a result, generation of incorrect lab results may occur which can lead to challenges with interpretation and treatment. Fortunately, spot urinalysis (protein(albumin)/creatinine ratio) using a first morning urine sample has been shown to generate results with comparable accuracy to 24‐h urine testing in different patient groups, including transplant patients [5]. Further, qualitative analysis of urine is of great importance as it gives evidence regarding the origin of the proteinuria (see **Types of proteinuria** below). Determining the fraction of proteins that are filtered by the glomerulus (like albumin, transferrin or immunoglobulin G) and proteins secreted by the tubules (such as alpha‐1‐microglobulin or beta‐2‐microglobulin) points to the altered part of the kidney graft.

The methods and thresholds for proteinuria/albuminuria are summarized in Table 1.

**TABLE 1:** Methods and thresholds for assessment of proteinuria (according to KDIGO guidelines, 2024).

Parameter	Collection method	Threshold for pathological finding
Proteinuria (total)	Spot urine	> 20 mg/mmol crea that is, > 200 mg/g crea (children ≥ 2 years) > 50 mg/mmol crea that is, > 500 mg/g crea (children 6–24 months) Nephrotic range: > 220 mg/mmol crea that is, > 2200 mg/g crea
	24‐h collection urine	> 96 mg/m^2^/day Nephrotic range: > 960 mg/m^2^/day
Albuminuria	Spot urine	> 3 mg/mmol crea that is, > 30 mg/g crea
Alpha‐1‐microglobulinuria Beta‐2‐microglobulinuria	Spot urine Spot urine	> 0.55 mg/mmol crea > 0.04 mg/mmol crea

*Note:* According to KDIGO clinical practice guideline for the care of kidney transplant recipients [6].

## 3 Prevalence of Post‐Transplant Proteinuria

The prevalence of post‐transplant proteinuria is different in various studies and ranges between 11% and 82% in children and adults [4, 7, 8, 9, 10, 11, 12, 13, 14, 15]. There are several reasons for this considerable variability, mostly because of the use of different cut‐offs for the definition of proteinuria used by various trialists.

In earlier adult trials using a less strict cut‐off of 2 g/day, the prevalence of proteinuria was reported to be 10% and 22% [7, 9]. In more recent trials using a stricter threshold that is similar to the threshold for native CKD patients (i.e., 0.15–0.3 g/day), the prevalence of proteinuria increased to 31%–45% [4, 13].

**FIGURE 1:**
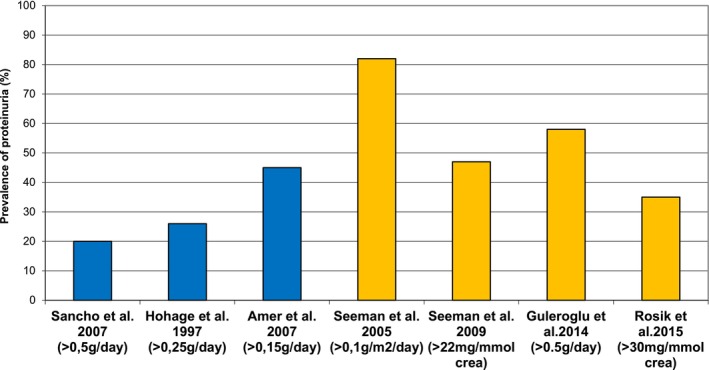
Prevalence of proteinuria in patients after kidney transplantation.

In pediatric kidney transplant recipients, proteinuria has been investigated in only a few studies [16, 17, 18, 19, 20, 21]. In one study, using a threshold of 20 mg/mmol creatinine to define proteinuria, 100% of children at 1 week post‐transplant had proteinuria [18]. In other studies, 47%–82% of patients, at least 6 months post‐transplant, using the thresholds of 4 mg/m^2^/day or 22 mg/mmol spot urine, were deemed to be proteinuric [17, 19]. In another study, proteinuria was detected in 58% of children at 3 months post‐transplantation when using a cut‐off of 500 mg/day [19]. The prevalence of proteinuria after kidney transplantation in different studies is shown in Figure 1.

## 4 Timing and Types of Post‐Transplant Proteinuria

### 4.1 Time of Onset and Persistence

Transient proteinuria is very common in the early post‐transplant period. Up to 100% of recipients show proteinuria at 1 week post‐transplant; however, in the vast majority of patients, this proteinuria regresses at the 2 month posttransplant follow up [17] and does not have deleterious effects on long‐term graft survival [7, 8, 9, 22]. Importantly, later onset proteinuria (2–3 months posttransplant) or persistent proteinuria (> 3 months duration) is associated with increased risk of long‐term graft loss [10, 23].

### 4.2 Glomerular and Tubular Types

Several studies have shown that the main type of proteinuria in patients after kidney transplantation is tubular. It has been detected in up to 79% of adults and in 80% of pediatric kidney transplant recipients [19, 22, 24, 25]. The more severe the proteinuria, the higher the likelihood it is of glomerular origin [25]. Glomerular‐type proteinuria with increased urinary excretion of albumin often signals glomerular injury such as recurrent or de novo glomerulopathies, transplant glomerulopathy (TG) or hypertension‐induced glomerulopathy of the allograft.

Based on the predominance of tubular proteinuria in transplant recipients over glomerular proteinuria, we suggest proteinuria should be measured in transplanted patients as total proteinuria (combined tubular and glomerular) that is captured in a 24 h urine collection as well as a surrogate first morning sample. Specifically, we suggest examining both glomerular (albuminuria) and tubular (e.g., alpha‐1‐microglobulin or beta‐2‐microglobulin) proteinuria.

### 4.3 Degree of Proteinuria

In cases of transplant proteinuria in children, the amount is usually borderline or mild, and non‐nephrotic (mean proteinuria in pediatric studies is approximately 200 mg/m^2^/day or 20 mg/mmol creatinine [16, 17, 19]).

In adults, the quantity of proteinuria is linked mainly to allograft glomerular pathology. Adults with post‐transplant nephrotic range proteinuria often have glomerular allograft injury (60%–80%, histologically mainly TG) compared to those with non‐nephrotic range proteinuria (12% glomerular injury) [9, 13, 25, 26].

From these studies it is apparent that both type (glomerular or tubular) and quantity (low grade, non‐nephrotic, nephrotic) of proteinuria are important to understanding the mechanism of graft injury.

## 5 Origin of Proteinuria

Proteinuria in transplanted patients could originate both from the allograft and from the remaining native kidneys. Importantly, often the native kidneys regress with time, thus lowering their impact on proteinuria analyses. Despite this, it is of great importance to know the origin of proteinuria, mainly in patients with a risk of disease recurrence such as idiopathic focal segmental glomerulosclerosis (FSGS) that most often presents in the first post‐transplant days as clinically significant nephrotic syndrome. A large trial on pre‐transplant proteinuric patients has shown that pre‐transplant proteinuria rapidly decreases as early as 3 weeks post‐transplant from 3.6 g/day (nephrotic range) to 0.5 g/day [27]. In a smaller study, similar results were shown with normalization of native kidney proteinuria in 100% of patients at an average time of 4 weeks post‐transplant [28]. It is apparent from these studies, that persistence of nephrotic range proteinuria 3–4 weeks post‐transplant should be considered from the allograft.

## 6 Causes and Risk Factors for Post‐Transplant Proteinuria

**FIGURE 2:**
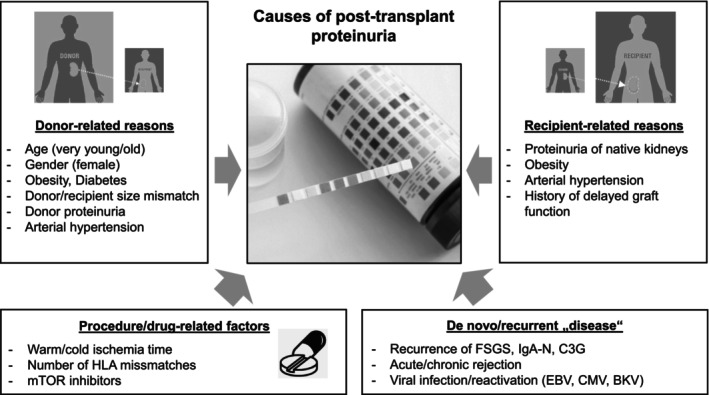
Causes of proteinuria in patients after kidney transplantation. C3G, C3 glomerulopathy; FSGS, focal segmental glomerulosclerosis; IgA‐N, IgA‐nephropathy; mTOR, mammalian target of rapamycin.

Etiology of post‐transplant proteinuria is diverse and may differ depending on the timing after transplantation, degree, or type of proteinuria. Post‐transplant proteinuria may be caused by existing recipient CKD (e.g., steroid‐resistant nephrotic syndrome) and obesity of the donor, as well as arterial hypertension of either patient or donor [13, 17, 29, 30]. Procedure‐related factors may also have an impact on proteinuria as a high number of donor‐recipient mismatches or a long cold or warm ischemia time are associated with higher rates of proteinuria [31]. Recurrence of immune‐mediated glomerular kidney disease of the recipient (like FSGS) is an important cause of high‐grade proteinuria and may occur immediately, within hours, or several days after transplantation. Acute (and later chronic) cellular and antibody‐mediated rejections are the leading causes of proteinuria [32] as are viral infections with the cytomegalovirus (CMV), Epstein–Barr (EBV) or BK polyoma viruses (BKV). Proteinuria can also be a drug‐specific side effect and is especially related to mTOR inhibitors [33]. The causes of post‐transplant proteinuria are depicted in Figure 2.

## 7 Clinical Consequences of Proteinuria in Transplant Recipients

### 7.1 Associations of Proteinuria With Graft Survival

#### 7.1.1 Adults

Proteinuria is a strong independent risk factor for graft loss [4, 8, 9, 10, 11, 12, 13, 14]. A large, nationwide Spanish study demonstrated that the presence of proteinuria at 1 year post‐transplant was a strong marker for impaired graft survival [12]. There is a correlation between the amount of proteinuria and the risk of graft loss beginning at even very low levels of proteinuria (0.15 g/day) [13, 15, 34]. Moreover, this correlation between proteinuria and graft survival, starting at as early as 3 months after transplantation, and lasting until one or more years post‐transplantation, remains significant [4, 12, 13, 15]. These study results are summarized in Table 2.

**TABLE 2:** Proteinuria, clinical, and allograft characteristics in kidney transplant recipients.

Author [reference]	Investigated subjects	Definition of proteinuria	Time after transplantation	Prevalence of proteinuria	Etiology/risk factors	Allograft/patient survival
Hohage et al. 1997 [10]	*n* = 357	> 0.25 g/day	1–6 months	26%	Increased serum creatinine	Impaired 5 year graft survival (59% vs. 86% in non‐proteinuric)
Roodnat et al. 2001 [4]	*n* = 722	> 0.2 g/L	1 year	33%	Primary renal diseases (GN, hypertension, systemic diseases)	Impaired graft and patient survival
Fernández‐Fresnedo et al. 2004 [12]	*n* = 3365	> 0.5 g/day	1 year	15%	Chronic rejection, primary renal disease, older donor age, DGF, acute rejection	Impaired graft and patient survival
Seeman et al. 2005 [17]	*n* = 33 (children)	> 0.1 g/m^2^/day	Different (mean 25 months)	82%	Ambulatory hypertension, history of acute rejection	n.d.
Sancho et al. 2007 [14]	*n* = 68	> 0.5 g/day	Different (mean 53 months)	20%	Increased serum creatinine, increased BP, DGF, BMI	Impaired 5 year graft survival (69% vs. 93% in non‐proteinuric)
Amer et al. 2007 [13]	*n* = 276 (protocol biopsies)	> 0.15 g/day	1 year	45%	Increased serum creatinine, increased BP	Impaired graft survival independent of other risk factors
Gulleroglu et al. 2014 [20]	*n* = 77 (children)	> 0.5 g/day	3 months	58%	No relationship with other outcomes parameter	n.d.
Seeman et al. 2009 [19]	*n* = 53 (children)	> 22 mg/mmol creatinine	4.3 years	47% (tubular in 80% of proteinuric pts)	Chronic allograft nephropathy	n.d.
Rosik et al. 2015 [23]	*n* = 75 (children)	> 30 mg/mmol creatinine	1 year	35% (recurrent FSGS pts. excluded)	Male sex, no treatment with ACEIs	Impaired 5 years graft survival in proteinuric children (77% vs. 100%)
Yilmaz et al. 2018 [21]	*n* = 52 (children)	Dipstick ≥ 1 or > 0.12 g/m2/day	1 month	65%	Proteinuria resolved during follow‐up in 59% of pts.	n.d.

Abbreviations: ACEIs, angiotensin‐converting enzyme inhibitors; BMI, body mass index; BP, blood pressure; DGF, delayed graft function; FSGS, focal segmental glomerulosclerosis; GN, glomerulonephritis; n.d., not determined; TG, transplant glomerulopathy.

Furthermore, proteinuria was found in several studies to be an independent risk factor for graft loss after adjustment for rejection and hypertension [4, 12, 31]. Both glomerular and tubular proteinuria were found to be associated with impaired graft survival [24, 25]. Despite these clear associations, it is still a matter of debate whether post‐transplant proteinuria is a real cause of graft loss or only the result of the primary pathology (e.g., recurrence of FSGS, acute and chronic rejection, hypertension, etc.) or both [35, 36, 37, 38].

#### 7.1.2 Children

In children with native CKD, increased urinary albumin and protein excretion is a significant risk factor for the progression of CKD [1, 2, 39]. Furthermore, antiproteinuric treatment with renin‐angiotensin‐aldosterone‐system (RAAS) blockers is associated with slower progression of CKD [39].

**FIGURE 3:**
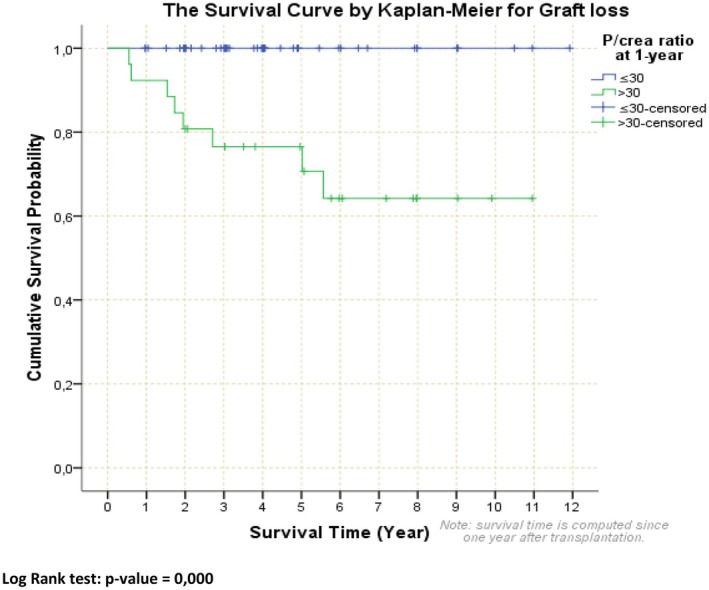
Graft survival following 1 year of proteinuria in pediatric kidney transplant recipients (with permission from Rosik et al. 2015 [23]). P/crea ratio protein/creatinine ratio.

In pediatric patients after kidney transplantation, only one single‐center retrospective study investigated the association between proteinuria and graft survival [23]. In a cohort of 75 children with proteinuria at 1‐year posttransplant (> 30 mg/mmol), there was a clear association with significantly worse 5‐year graft survival (77%) compared to children with minimal proteinuria (< 30 mg/mmol; 100%, Figure 3). Furthermore, proteinuria at 1‐year posttransplant together with acute rejection was revealed to be the only independent predictor of graft loss. Importantly, there is an ongoing study on the association between proteinuria and graft survival within the large, multicenter CERTAIN registry.

Studies that have investigated graft survival in children with recurrence of proteinuria as a result of their primary disease (idiopathic FSGS) showed conflicting results. Some studies showed similar graft survival with non‐FSGS patients [40, 41], whereas others demonstrated worse graft survival in those with recurrence [42, 43]. The reason for this discrepancy is best explained by the different percentage of patients reaching remission of proteinuria with therapy (no remission with long‐lasting proteinuria is strongly associated with poor graft survival). Altogether, it seems that proteinuria is associated with graft loss and lower graft survival in children similarly to adults. The pediatric studies on posttransplant proteinuria are summarized in Table 2.

## 8 Associations of Proteinuria With Patient Survival

### 8.1 Adults

Proteinuria in adult transplanted patients, similar to the general adult population, is associated with higher patient mortality [4, 12].

Similar studies in children are scarce as overall mortality is lower in the pediatric population. One retrospective single‐center study showed patient survival in proteinuric pediatric patients was similar to non‐proteinuric patients [23].

## 9 Diagnostics of Post‐Transplant Proteinuria

In order to effectively manage and treat post‐transplant proteinuria, the underlying etiology must be determined (e.g., recurrence, rejection).

### 9.1 Urinary Tests

The current 2024 Kidney Disease Improving Global Outcomes (KDIGO) clinical practice guideline on the care of adult transplant recipients [6] suggests measuring proteinuria at least once within the first month after transplantation, at least every 3 months between months 1 and 12, and at least annually ≥ 1‐year post‐transplant.

In children, we suggest screening for proteinuria on a regular basis during long‐term clinical posttransplant care. Urinary dipstick is sufficient to identify new‐onset (higher grade) proteinuria and should be part of every outpatient visit. If the dipstick reveals new‐onset proteinuria or an increase in pre‐existing proteinuria, it should be quantified. Protein (albumin)/creatinine ratio using spot urine should be performed at least every 1 to 3 months. In all other children with negative dipstick, quantitative assessment of proteinuria should be performed at least every 6 to 12 months. A 24 h urine collection for protein excretion is not necessary if the patient does not suffer from reduced muscle mass (e.g., due to general muscle wasting or myopathy) which may lead to reduced creatinine in blood and urine.

Urinary proteomic analysis is a promising tool to detect prognostic biomarkers (e.g., monocyte chemoattractant protein‐1) associated with different causes of graft proteinuria and graft failure. This emerging field is of great interest as it promotes a non‐invasive method to further classify graft damage, a so‐called ‘liquid biopsy’ which may replace the current gold standard renal biopsy in the future. However, much investigation and scrutiny remain before this is used in clinical practice.

Special care must be taken in patients with idiopathic FSGS who have a high risk of recurrence. In these patients, proteinuria should be measured daily in the first 2–3 weeks post‐transplant to detect and treat possible recurrence as early as possible. Thereafter, proteinuria should be measured at every outpatient visit during the first year post‐transplant as recurrence can occur also later.

### 9.2 Blood Tests, Imaging Studies and Graft Biopsy

In addition to urinary diagnostic testing, blood tests to assess the impact of proteinuria on graft function and to evaluate its secondary effects, for example, diminished serum proteins important for coagulation and thyroid function, are mandatory. Blood tests can also unravel important causes of proteinuria like viral infections (by detection of viral DNA of CMV, EBV, or BKV) or antibody‐mediated rejection (via detection of donor‐specific antibodies). Doppler ultrasonography of the transplanted kidney and, as mentioned before, a graft biopsy are further required to thoroughly evaluate graft pathology.

Renal graft biopsy is not always necessary before initiation of treatment in suspected recurrence of idiopathic FSGS with acute onset nephrotic‐range proteinuria. Contrarily, the biopsy should be performed in unexplained persistent proteinuria (> 3 months duration), especially in new‐onset proteinuria, acute kidney injury, acute deterioration of graft function, or unexplained nephrotic‐range proteinuria. This is especially important as these patients tend to show graft‐specific pathological findings such as de novo glomerulonephritis or transplant glomerulopathy [27]. Other common renal pathology findings associated with proteinuria after kidney transplantations are acute or chronic antibody‐mediated rejection, TG, acute T‐cell mediated rejection, recurrence of FSGS or IgA nephropathy [13].

**FIGURE 4:**
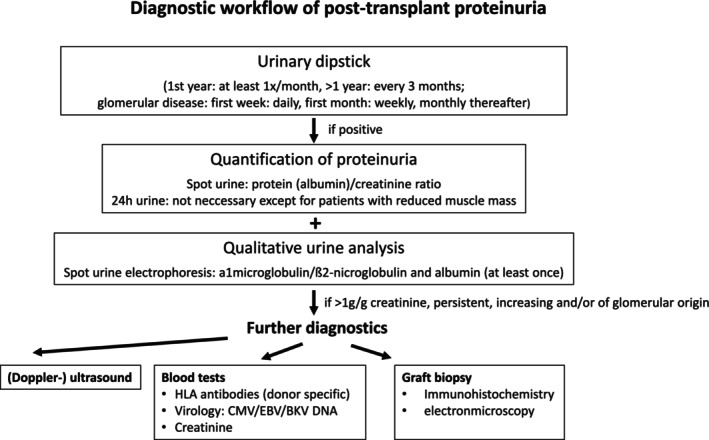
Diagnostic algorithm for a transplanted child with proteinuria. ABPM, ambulatory blood pressure monitoring; BP, blood pressure; FSGS, focal segmental glomerulosclerosis; HT, hypertension.

The diagnostic algorithm for proteinuria in transplanted children is given in Figure 4.

## 10 Treatment of Post‐Transplant Proteinuria

Therapy for post‐transplant proteinuria should be started soon after its diagnosis, but the clinician should keep the following considerations in mind:

– Persistent proteinuria (> 3 months duration) of any degree (> 20 mg/mmol creatinine, or 200 mg/day) will require therapy [6, 44]. However, early proteinuria (during the first 3 months post‐transplant) is mostly transient [18], unless caused by recurrence of glomerular diseases such as FSGS.
– Unexplained nephrotic‐range proteinuria of any duration and any time after transplantation requires prompt intervention.

Interventions and treatment for proteinuria should be directed at the underlying cause whenever possible and based on symptoms whenever a clear cause cannot be identified.

### 10.1 Causal Treatment

If the cause of post‐transplant proteinuria can be identified, targeted therapy should be initiated, and if appropriate, anti‐proteinuric therapy via blockade of RAAS. Depending on the underlying pathology, an intensification of immunosuppressive therapy may be necessary, for example, in cases of cellular or antibody‐mediated rejection or the recurrence of a primary chronic kidney disease (e.g., FSGS or IgA‐nephropathy). The most promising treatments of recurrent FSGS post‐transplant are early plasma exchange or immunoadsorption with or without high‐dose cyclosporine‐A and/or rituximab that can often induce remission of nephrotic syndrome [45].

In case of allograft rejection, treatments may include plasmapheresis, steroid pulse therapy, or monoclonal anti‐CD20 antibodies and an increase in basal triple immunosuppression (tacrolimus, mycophenolate mofetil and prednisolone). On the contrary, a viral infection, such as BKV, may require a reduction of immunosuppression or the replacement of mycophenolate mofetil by an mTOR inhibitor. An infection with CMV can be treated with valganciclovir. If proteinuria is considered to be an mTOR inhibitor related side effect, it should be replaced by other antiproliferative medication (like mycophenolate mofetil).

### 10.2 Symptomatic Treatment

#### 10.2.1 Non‐Specific Antiproteinuric Treatment With Angiotensin‐Converting Enzyme Inhibitors (ACEI) and Angiotensin Receptor Blockers (ARBs)

ACEI and ARBs decrease proteinuria in adults and children after kidney transplantation [46, 47]. In a randomized controlled trial in adults following kidney transplantation who had proteinuria, ramipril reduced proteinuria significantly compared to placebo. Additionally, it decreased the renal end point of doubling of serum creatinine by 65%, and graft loss by 55%, but it did not significantly decrease the composite primary endpoint (doubling of serum creatinine, return to dialysis, death) [48]. Therefore, in adults, it seems that ACEIs are associated with reduced graft loss but not with reduced mortality.

In one prospective single‐center study, ramipril reduced proteinuria in about 90% of proteinuric children and this antiproteinuric effect was seen without a BP lowering effect [47]. Therefore, ACEI/ARB should be used in proteinuric children with, but also without, hypertension. This is in line with the recent KDIGO guidelines on blood pressure in CKD [49]. Antiproteinuric treatment with ACEI/ARB should be initiated when proteinuria is persistently (> 3 months) > 20 mg/mmol creatinine. This is based on the KDIGO recommendation for non‐transplanted children with CKD and proteinuria [44]. The doses that should be used for antiproteinuric treatment are similar to children with chronic native kidney diseases.

The dual inhibition of the RAAS using both ACEIs and ARBs in combination is able to further decrease urinary protein excretion by 30%–40% in pediatric patients with native CKD already on maximal doses of ACEI [50, 51]. Similar dual RAAS blockade is also possible in transplanted patients [19] but caution must be taken due to increased risks of hyperkalemia or acute graft dysfunction, especially if a child is dehydrated. During such situations both ACEI and ARB should be temporarily discontinued [44].

The mechanisms by which ACEI/ARB reduce proteinuria are multiple, and include: hemodynamic mechanisms, like reduction of arterial as well as intraglomerular pressure, and non‐hemodynamic mechanisms, like antiproliferative and antifibrotic effects, and preservation of the podocyte slit diaphragm structure [52].

### 10.3 Blood Pressure Lowering Therapy Beyond RAAS‐Inhibitors

#### 10.3.1 Target BP for Proteinuric Children Post‐Transplant

It is well understood that following solid organ transplantation in children, particularly kidney transplantation, there can be an elevated blood pressure (BP) related to not only the graft, but also medications, or fluid overload [53, 54]. Further, many children will be hypertensive prior to their transplant, which is another consideration for antihypertensive therapy [55].

Significant evidence exists suggesting that hypertension in post‐transplant patients may lead not only to graft failure but also increases in cardiovascular morbidity [56]. As such, much has been done to try to identify a target BP for those children post‐transplant; however, to date, there is no good evidence to suggest an appropriate target. At present, most recommendations are for children post‐transplant to have a BP < 95th percentile, similar to healthy children [56]. For those children post‐transplant who are proteinuric, there is even less evidence as to what an established target BP should be. It has been described that children with hypertension who are post‐transplant are more likely to have proteinuria, and as such, an obvious recommendation would be to intervene to address this hypertension. The recommendation of the current KDIGO guidelines for blood pressure management in kidney transplant recipients [49], KDIGO guidelines for kidney transplant recipients [6], the European Society for Hypertension pediatric guidelines, and the American Academy of Pediatrics Clinical Practice Guidelines for CKD in children recommend targeting BP < 50th percentile [57, 58]. However, it should be noted that no study in adults or children has ever shown a benefit of these strict BP targets in transplanted patients. Moreover, strict BP control (BP < 50th percentile) did not lower proteinuria in the ESCORT trial [59] but seemed to induce regression of left ventricular hypertrophy [60]. The most recent KDIGO guidelines for BP management in kidney transplant recipients [49] state that, in contrast to the general population, a lower systolic BP target such as < 120 mmHg may not be appropriate for kidney transplant recipients without further data on the risks and benefits of targeting lower BP levels in the transplant population. Therefore, BP targets similar to the general pediatric population may be reasonable for proteinuric transplanted children. In light of this, there is clearly an unmet need for larger interventional trials in pediatric kidney transplant recipients [42, 46, 56, 61].

For those resistant to this standard antiproteinuric therapy, addition of alternative antihypertensives is recommended to achieve a stricter target BP (including calcium channel blockers, diuretics, and beta blockers). Antiproteinuric effects of non‐ACEI/ARB antihypertensives could also be demonstrated in pediatric transplant patients with proteinuria. In one single‐center prospective study, proteinuria was lowered with a reduction in BP using only non‐ACEI/ARB antihypertensives [7, 8, 62].

### 10.4 New Antiproteinuric Agents

#### 10.4.1 Mineralocorticoid Receptor Antagonists

Mineralocorticoid receptor antagonists (MRA) block aldosterone's action at the mineralocorticoid receptor, mitigate the deleterious effects of aldosterone‐driven oxidative stress, inflammation, fibrosis, and vascular damage [63, 64].

Initial data from transplanted patients indicate that MRA effectively reduces proteinuria, ischemia–reperfusion injury, and calcineurin inhibitor nephrotoxicity, with minimal impact on glomerular filtration rate and no significant rise in the risk of hyperkalemia [65]. In the intermediate and nephrotic range groups, spironolactone therapy resulted in a decrease in proteinuria after 6 months of treatment, which continued for a full year of follow‐up without causing a discernible drop in estimated glomerular filtration rate. Gonzales‐Monte et al. showed a sustained > 50% reduction in proteinuria (mean 81%) after the addition of spironolactone with only mild, nonsignificant renal function decline [66]. Baskin et al. demonstrated that in pediatric kidney transplant patients with chronic allograft nephropathy and significant proteinuria, eplerenone lowered proteinuria and maintained stable glomerular filtration rate [67]. These findings suggest that MRAs can be safely used in selected transplant patients with proper monitoring [68].

#### 10.4.2 Sodium‐Glucose Cotransporter‐2‐Inhibitors

Sodium‐glucose cotransporter‐2 inhibitors (SGLT2i) have shown impressive potential in cardio‐renal protection in patients with heart failure [69, 70] and CKD in both diabetic and non‐diabetic, non‐transplanted CKD patients [71, 72]. For this reason, the use of SGLT2i has been included in the KDIGO 2024 recommendations for the management of patients with proteinuric and non‐proteinuric CKD [6]. Several studies reported the safety of SGLT2i use in adult transplanted patients, mainly after heart and kidney transplant, in both diabetic and non‐diabetic patients, showing low infection rates and stable immunosuppression levels [73, 74]. The FDA and EMA approved the use of SGLT2i in children older than 10 years suffering from Type 2 diabetes. Multiple studies have now demonstrated the safe use of SGLT2i in diabetic and non‐diabetic children [75, 76]. The antihypertensive and antiproteinuric effects observed in adults were confirmed in non‐transplanted children [77]. Nevertheless, trials evaluating the safety and efficacy of SGLT2i in children post‐transplant are needed.

#### 10.4.3 Vitamin D‐Receptor Analogues

Research with animal models suggests that vitamin D supplementation may reduce proteinuria through direct protective effects on podocytes, or through various pleiotropic mechanisms that reduce RAAS activation, inflammation, and fibrosis [78]. Multiple investigations have shown substantial kidney‐protective effects of active vitamin D (1,25‐dihydroxycholecalciferol) across various kidney disease etiologies [79]. However, research involving kidney transplant recipients has produced inconsistent findings [80, 81]. Currently, there are no prospective randomized studies examining the relationship between proteinuria and vitamin D supplementation in pediatric kidney transplant recipients. While vitamin D analogs may serve as supplementary treatment for proteinuria, further research is needed to determine whether this approach improves transplant outcomes.

#### 10.4.4 Endothelin Receptor Antagonist

Endothelin is implicated in the pathogenesis of kidney fibrosis and inflammation, both of which exacerbate proteinuria. Endothelin (ET) levels increase after graft transplantation and may play a significant role in the development of post‐transplant complications [82]. Sparsentan is a dual‐action angiotensin II type 1 receptor antagonist and endothelin receptor A (ETA) antagonist that exerts potent antiproteinuric effects. Clinical studies have demonstrated that sparsentan effectively reduces proteinuria in patients with chronic kidney diseases [83]. Sparsentan has not been extensively studied in kidney transplant recipients, and its use in this patient population is limited.

## 11 Target Proteinuria in Transplanted Children

**FIGURE 5:**
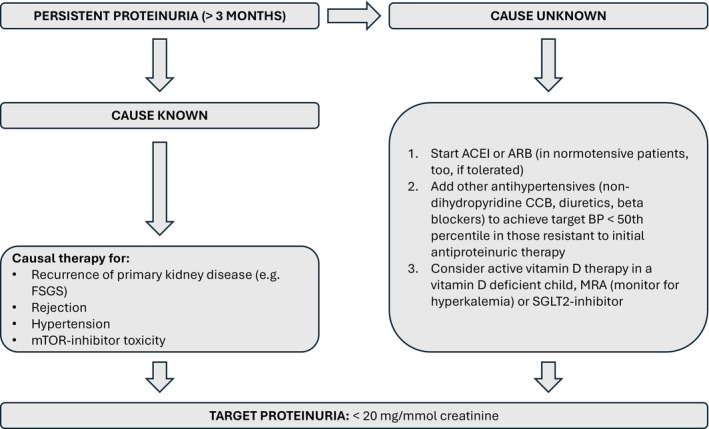
Treatment algorithm for a transplanted child with persistent proteinuria. ACEI, angiotensin‐converting enzyme inhibitor; ARB, angiotensin receptor blocker; BP, blood pressure; CCB, calcium channel blocker; FSGS, focal segmental glomerulosclerosis.

Currently, no guidelines clearly state what the target for acceptable proteinuria should be in kidney transplant recipients. Taking into account the evidence that even low‐level proteinuria (0.15–0.2 g/day or 25–30 mg/mmol) is associated with impaired graft survival in adults [14, 40], and that children with proteinuria > 30 mg/mmol have worse graft survival than those < 30 mg/mmol creatinine [23], and that the threshold for normal proteinuria according to the recent KDIGO guidelines for native CKD is < 20 mg/mmol [44], we suggest that the target proteinuria levels should be < 20 mg/mmol in children post‐transplant, as long as treatment is well tolerated. The treatment algorithm for proteinuria in transplanted children is given in Figure 5.

## 12 Can Decreasing Proteinuria Improve Graft Survival?

### 12.1 Adults

There is only one randomized, placebo‐controlled, 4‐year trial investigating the effects of antiproteinuric therapy with ramipril, as discussed above [48]. There was a 33% reduction in graft failure in the ramipril group but without any positive effect of ramipril on the composite primary endpoint including patient survival.

### 12.2 Children

While it is clear that hypertension can lead to poor graft survival, it is less clear whether treatment with antiproteinuric agents, and effective decreasing of proteinuria, can improve kidney graft survival in children. The only prospective interventional trial investigating ACEi (ramipril) for 6 months in proteinuric children showed antiproteinuric efficacy of ramipril but due to the short duration of the trial, long‐term graft survival could not be evaluated [47]. A retrospective study on ACEIs in children post‐transplant showed again their antiproteinuric effect but this study also did not assess graft survival [46]. Therefore, large, prospective, controlled, pediatric trials are needed to investigate possible nephroprotective effects of antiproteinuric therapy.

## 13 Areas for Further Research

In the future, research should concentrate on elucidating whether:

– Anti‐proteinuric treatment can improve graft function and graft survival in children.
– A strict proteinuria target (< 20 mg/mmol) is associated with better graft survival compared to the conservative target (< 30 mg/mmol).
– Proteinuria in transplanted children is associated with decreased patient survival, and if so, can new antiproteinuric treatments which have a safe profile in transplanted children be used to mitigate this risk.

## 14 Conclusions

Proteinuria is a frequent complication in pediatric kidney transplant recipients (40%–80%). The most common etiologies and risk factors are recurrent FSGS, rejection, mTOR‐inhibitors and hypertension. Proteinuria after transplantation is, independently of its cause, associated with impaired graft survival in both adults and children and is an important independent prognostic factor for graft loss. Proteinuria should be investigated routinely in all transplanted children, and when discovered, should be treated early. Therapy for proteinuria in transplanted patients should be focused primarily on the etiology. Currently, the only validated symptomatic antiproteinuric treatment post‐transplantation consists of RAAS blockers.

## Author Contributions

T.S. prepared the design of the manuscript and participated in drafting the article; R.L.M., J.F., M.B., R.S.D., D.L.E., and A.B. participated in drafting the article.

## Funding

This work was supported by the Conceptual Development of the Research Organization, MH CZ‐DRO‐FNOs/2023.

## Conflicts of Interest

The authors declare no conflicts of interest.

## Data Availability

Data sharing not applicable to this article as no datasets were generated or analysed during the current study.
